# Quality of Life Assessment in Heart Failure Patients: Insights from a Low- to Middle-Income Country

**DOI:** 10.5334/gh.1538

**Published:** 2026-03-17

**Authors:** Muhammad Nauman Khan, Najia Aslam Soomro, Muhammad Navaid Iqbal, Rubina Rauf, Hina Sohail, Khalid Naseeb

**Affiliations:** 1 National Institute of Cardiovascular Diseases, Karachi, Pakistan; 2 Liaquat National Hospital, Karachi, Pakistan

**Keywords:** heart failure, quality of life, KCCQ-12, medication non-adherence

## Abstract

**Background:**

Heart failure (HF) continues to have an immense impact, not only on tangible outcomes such as mortality, but also on patients’ health-related quality of life (QoL). The aim of this study was to assess the QoL of patients living with HF using the Kansas City Cardiomyopathy Questionnaire (KCCQ-12).

**Methods:**

The study considered consecutive patients of either gender, between 18 and 80 years of age, who had been diagnosed with HF with reduced ejection fraction for at least six months and who presented to the outpatient department for routine clinical follow-up. The QoL was assessed using KCCQ-12.

**Results:**

A total of 320 patients were included in this analysis; 257 (80.3%) were male, and the mean age was 54.9 ± 12.7 years, with 25 (7.8%) patients >70 years. The median time since HF was 24 (12–60) months. The median overall QoL score was 50.5 (32–75), with 81 (25.3%) categorized as good, 83 (25.9%) as moderate, 99 (30.9%) as poor, and 57 (17.8%) as having very poor QoL. In multivariable analysis, age >70 years, presence of diabetes, and non-adherence to medication were found to be independently associated with poor QoL, with adjusted odds ratios of 2.93 [1.06–8.06], 2.76 [1.66–4.61], and 1.72 [1.07–2.77], respectively.

**Conclusion:**

We observed good QoL in only one-fourth of the patients living with HF. Further, we observed that older age (>70 years), presence of diabetes, and non-adherence to medication were significantly associated with poorer QoL. These results underscore the need for targeted interventions to improve medication adherence and manage comorbidities, particularly diabetes, in order to enhance the QoL of HF patients.

## Introduction

Heart failure (HF) is a chronic condition that greatly impacts patients’ quality of life (QoL). It not only affects tangible outcomes such as mortality (1), but also influences how patients perceive their health and well-being (2). The negative effects of HF on health-related quality of life (HR-QoL) are well recognized. Although new treatments have improved survival rates in recent years, many patients continue to experience severe symptoms in the later stages of the disease, emphasizing the need for a comprehensive approach to care (3).

Improving HR-QoL is a key goal in HF management, as highlighted by guidelines from North America, Europe, and the UK (4,5). Understanding patients’ perspectives on how HF affects their health, including their symptoms, daily functioning, and overall QoL, is becoming increasingly important in clinical trials, quality assessments, and everyday care (6). Over the past 30 years, several disease-specific tools for measuring patient-reported outcomes in HF have been developed (7). From the start, QoL questionnaires in HF have shown that QoL is influenced by multiple factors, including age, gender, New York Heart Association (NYHA) functional class, hospitalizations, frailty, and newer treatments (8).

One widely used tool for assessing QoL in HF patients is the Kansas City Cardiomyopathy Questionnaire (KCCQ). It is known for its reliability, validity, and sensitivity. However, its length has sometimes limited its practicality. To address this, Spertus et al. developed a shorter version, KCCQ-12, which retains the strengths of the original but is more practical for routine use (7).

HF has a greater impact on QoL than many other chronic diseases (9). Despite this, clinical management and research often do not pay sufficient attention to this aspect in our population. Therefore, in this study, we aimed to assess the QoL of patients living with HF using KCCQ-12. While several studies, including large international registries, have examined QoL in heart failure patients, most have originated from high-income countries, with limited representation from South Asia, particularly Pakistan (1). Given Pakistan’s unique healthcare challenges, resource constraints, and cultural influences on treatment adherence and self-perception of health, local data are essential to inform effective patient-centered interventions. This study seeks to fill this gap by providing insights into QoL among HF patients in a Pakistani tertiary care setting using a validated Urdu translation of KCCQ-12.

## Materials and Methods

### Study design

This descriptive cross-sectional study was conducted at the outpatient department of the National Institute of Cardiovascular Diseases (NICVD), the largest public-sector cardiac care hospital in Karachi, Pakistan. This study aimed to assess the QoL of patients living with heart failure who presented to the outpatient department between October 2019 and March 2020.

### Ethics

The study was conducted in accordance with the guidelines provided by the Declaration of Helsinki. Prior to data collection, the study proposal was presented to the institutional review board of the NICVD, and it was approved with reference number ERC-49/2019. Additionally, the data collection process was explained to all participants, and informed consent for the study was documented for all participants.

### Study population

The study population consisted of a consecutive sample of patients of either gender, between 18 and 80 years of age, who presented to the outpatient department, had been diagnosed with heart failure with reduced ejection fraction (HFrEF) for at least six months, and who visited the outpatient heart failure clinic for routine follow-up. Patients who refused to participate in the study or those with chronic kidney disease, congenital heart disease, psychiatric illness, or any other health condition leading to restricted mobility were excluded from the study.

### Data collection

Data for the study were collected using a predefined structured proforma. Collected data included demographic information such as age; gender; duration of heart failure; comorbid conditions such as diabetes, hypertension, and dyslipidemia; smoking; heart failure medications; and QoL. Assisted interviews were conducted with all patients to assess QoL.

### Variables and definitions

Heart failure was diagnosed based on clinical symptoms (e.g., dyspnea, fatigue, edema), signs (e.g., elevated jugular venous pressure, rales), and echocardiographic evidence of reduced left ventricular ejection fraction (LVEF ≤40%) as per the 2016 European Society of Cardiology guidelines. Only patients with a confirmed diagnosis of HFrEF for at least six months were included. Comorbid conditions such as diabetes, hypertension, and dyslipidemia were defined based on documented medical history and records indicating that the patient was receiving treatment for the respective condition. Smoking status was determined based on self-reported current or past tobacco use.

QoL was assessed using the Urdu translation of the short version of the validated KCCQ-12 (7). Permission to use KCCQ-12 was requested from the CV Outcomes website (https://cvoutcomes.org), and a license to use the validated Urdu translation of the questionnaire was purchased.

QoL was summarized into four domains, namely ‘Physical Limitation’, ‘Symptom Frequency’, ‘Quality of Life’, and ‘Social Limitation’, along with an overall summary score. The summary scores were rescaled to 0 to 100 and categorized as very poor (<25), poor (26 to <50), moderate (50 to <75), and good (≥75).

Medication non-adherence was assessed using the validated DOSE-Non-adherence questionnaire (10). Non-adherent individuals were defined as those who provided a rating >1 for any of the three Extent of Non-adherence items.

### Sample size

In the absence of an estimate of QoL for our local population, the sample size was calculated with an expected rate of 20% having a good QoL, at a 95% confidence level, and with a margin of error of 5%. The sample size for the study was calculated to be 246 patients. To account for random and sampling error, 30% additional patients were required; hence, the final sample size for the study was 320 patients with HF.

### Data analysis

The collected data were summarized in accordance with the study objective. Appropriate summary measures such as mean ± standard deviation or median [interquartile range] were computed for age, duration of heart failure, and QoL. The distributions of various clinical and demographic variables, along with outcome variables, were expressed as percentages (%). Patients were categorized into two groups based on overall QoL summary score, with a cutoff value of ≥50 considered good QoL. The appropriate t-test/Mann–Whitney U test or Chi-square test/Fisher’s exact test was applied. Multi-variable binary logistic regression analysis with backward variable selection was performed to identify factors associated with poor QoL of patients living with heart failure. Adjusted odds ratios (ORs) and 95% confidence intervals (CIs) were computed with the significance level set at p ≤ 0.05.

## Results

A total of 320 patients were included in this analysis; 257 (80.3%) were male, and the mean age was 54.9 ± 12.7 years, with 25 (7.8%) patients >70 years. The median time since heart failure was 24 (12–60) months. Non-adherence to medication was observed in 136 (42.5%) patients. The median overall summary QoL score was 50.5 (32–75), with 81 (25.3%) categorized as good, 83 (25.9%) as moderate, 99 (30.9%) as poor, and 57 (17.8%) as having very poor QoL (Table 1).

**Table 1:** Distribution of clinical characteristics and QoL of patients with heart failure.


	TOTAL

**Total (N)**	**320**

**Gender**	

Male	257 (80.3%)

Female	63 (19.7%)

**Mean Age (years)**	54.9 ± 12.7

≤50 years	109 (34.1%)

51 to 70 years	186 (58.1%)

>70 years	25 (7.8%)

**Median months since heart failure**	24 (12–60)

1 year or less	104 (32.5%)

1 to 3 years	101 (31.6%)

More than 3 years	115 (35.9%)

**Comorbid conditions**	

Diabetics	141 (44.1%)

Hypertensive	206 (64.4%)

Dyslipidemia	116 (36.3%)

Smokers	111 (34.7%)

**Heart failure medication adherence status**	

Adherent	184 (57.5%)

Non-adherent	136 (42.5%)

**Median Physical Limitation Score**	50 (25–75)

Very Poor (<25)	51 (15.9%)

Poor (25 to <50)	80 (25%)

Moderate (50 to <75)	94 (29.4%)

Good (>75)	95 (29.7%)

**Median Symptom Frequency Score**	63 (38–92)

Very Poor (<25)	37 (11.6%)

Poor (25 to <50)	73 (22.8%)

Moderate (50 to <75)	76 (23.8%)

Good (>75)	134 (41.9%)

**Median Quality of Life Score**	50 (25–75)

Very Poor (<25)	55 (17.2%)

Poor (25 to <50)	67 (20.9%)

Moderate (50 to <75)	92 (28.8%)

Good (>75)	106 (33.1%)

**Median Social Limitation Score**	42 (17–75)

Very Poor (<25)	80 (25%)

Poor (25 to <50)	78 (24.4%)

Moderate (50 to <75)	68 (21.3%)

Good (>75)	94 (29.4%)

**Median Overall Summary score**	50.5 (32–75)

Very Poor (<25)	57 (17.8%)

Poor (25 to <50)	99 (30.9%)

Moderate (50 to <75)	83 (25.9%)

Good (>75)	81 (25.3%)


Compared to patients with good QoL, patients with poor QoL were older, with a mean age of 56.9 ± 13.2 years vs. 53 ± 11.9 years; p = 0.006. Non-adherence to medication was found to be positively associated with poor QoL among these patients (Table 2).

**Table 2:** Association of clinical characteristics and QoL of patients living with heart failure.


	TOTAL	SUMMARY SCORE		P-VALUE

		POOR (<50)	GOOD (≥50)	

**Total (N)**	**320**	**156**	**164**	–

**Gender**				

Male	257	119 (46.3%)	138 (53.7%)	0.077

Female	63	37 (58.7%)	26 (41.3%)	

**Mean Age (years)**	54.9 ± 12.7	56.9 ± 13.2	53 ± 11.9	0.006

≤50 years	109	46 (42.2%)	63 (57.8%)	0.026

51 to 70 years	186	92 (49.5%)	94 (50.5%)	

>70 years	25	18 (72%)	7 (28%)	

**Median months since heart failure**	24 (12–60)	30 (10–60)	24 (12–48)	0.903

1 year or less	104	53 (51%)	51 (49%)	0.319

1 to 3 years	101	43 (42.6%)	58 (57.4%)	

More than 3 years	115	60 (52.2%)	55 (47.8%)	

**Diabetes mellitus**				

Non-diabetics	179	71 (39.7%)	108 (60.3%)	<0.001

Diabetics	141	85 (60.3%)	56 (39.7%)	

**Hypertension**				

Non-hypertensive	114	63 (55.3%)	51 (44.7%)	0.083

Hypertensive	206	93 (45.1%)	113 (54.9%)	

**Dyslipidemia**				

Non-dyslipidemia	204	108 (52.9%)	96 (47.1%)	0.047

Dyslipidemia	116	48 (41.4%)	68 (58.6%)	

**Smoking**				

Non-smokers	209	104 (49.8%)	105 (50.2%)	0.620

Smokers	111	52 (46.8%)	59 (53.2%)	

**Heart failure medication adherence status**				

Adherent	184	78 (42.4%)	106 (57.6%)	0.008

Non-adherent	136	78 (57.4%)	58 (42.6%)	


Following multivariable binary logistic regression analysis, age >70 years, presence of diabetes, and non-adherence to medication were found to be associated with poor QoL, with adjusted ORs of 2.93 [95% CI: 1.06–8.06; p = 0.037], 2.76 [95% CI: 1.66–4.61; p < 0.001], and 1.72 [95% CI: 1.07–2.77; p = 0.026], respectively (Table 3).

**Table 3:** Multi-variable binary logistic regression analysis for poor QoL of patients living with heart failure.


CHARACTERISTICS	INITIAL SOLUTION		FINAL SOLUTION	
	
	OR [95% CI]	P-VALUE	OR [95% CI]	P-VALUE

**Female**	1.78 [0.95–3.36]	0.074	1.75 [0.96–3.17]	0.066

**Age (years)**				

≤50 years (reference)	1	–	1	–

51 to 70 years	1.1 [0.64–1.88]	0.740	1.09 [0.64–1.86]	0.746

>70 years	2.75 [1–7.56]	0.050	2.93 [1.06–8.06]	0.037

**Months since heart failure**				

1 year or less (reference)	1	–	–	–

1 to 3 years	0.61 [0.33–1.11]	0.107	–	–

More than 3 years	0.89 [0.5–1.58]	0.689	–	–

**Diabetes mellitus**	2.86 [1.7–4.8]	<0.001	2.76 [1.66–4.61]	<0.001

**Hypertension**	0.54 [0.32–0.9]	0.018	0.53 [0.32–0.89]	0.015

**Dyslipidemia**	0.61 [0.37–1.02]	0.057	0.64 [0.39–1.05]	0.077

**Smoking**	0.89 [0.53–1.51]	0.672	–	–

**Non-adherence to HF medications**	1.72 [1.06–2.78]	0.028	1.72 [1.07–2.77]	0.026


## Discussion

This study provides real-world insight into the health-related QoL among patients with heart failure in a lower-middle-income country, using a validated local version of KCCQ-12. We observed that only 25.3% of patients reported good QoL, while nearly half experienced poor or very poor QoL, with an overall median QoL score of 50.5, highlighting a significant burden.

Age over 70 years, diabetes mellitus, and non-adherence to medication were all independently associated with poor QoL in multivariable analysis. Specifically, patients over 70 were nearly three times more likely to have poor QoL (OR: 2.93), those with diabetes had more than twice the risk (OR: 2.76), and those not adhering to their medication regimen were also at higher risk (OR: 1.72). While the link between older age, additional health conditions, and poorer QoL has been shown in other studies, our finding regarding medication non-adherence is novel.

Our findings suggest that medication adherence is associated with better QoL among heart failure patients. However, due to the cross-sectional nature of our study, we cannot determine the directionality of this relationship. Patients with lower QoL may also be less adherent due to medication side effects or a greater symptom burden. Future prospective and interventional studies are needed to evaluate whether improving adherence can causally improve QoL.

In addition to the factors highlighted in our study, QoL is a subjective assessment affected by multiple factors, such as treatment, previous hospitalizations, comorbidities such as diabetes, sex, and age (11,12). Undeniably, the physical dimension is also important in the QoL assessment; it is not surprising that QoL was reported to be poorer in patients with higher NYHA functional classes or more comorbidities (13). Past studies have shown that an improvement in the NYHA functional class translated into a favorable impact on QoL (11). In a study by Roy et al. (14), financial status, physical health, daily living independence, and resilience were linked to QoL, explaining 53% of its variance. Similarly, Gallagher et al. (15) reported that HR-QoL scores correlated significantly with NYHA class but showed wide variability within each class. No significant associations were found between HR-QoL scores and patient demographics, left ventricular ejection fraction, plasma BNP, or renal function. Furthermore, Ewnetu Tarekegn et al. (16) reported poor physical HR-QoL and identified significant factors influencing QoL in HF patients, including age, residence, marital status, income, and HF duration. Similarly, in a study by Zhang et al. (17), social support, self-efficacy, self-care management, monthly income, and residence were reported to be key factors influencing QoL. Abdi et al. (18) showed a significant positive correlation between spiritual well-being and both QoL and life expectancy. In a systematic review conducted by Kyriakou et al. (19), a positive but not statistically significant effect of social support on HR-QoL was found. However, significant positive effects were found for the physical and emotional support dimensions.

Improving QoL for HF patients often involves reducing symptoms such as edema, fatigue, and shortness of breath, as well as increasing patients’ ability to exercise (20). Symptoms such as edema, difficulty breathing, and reduced ability to perform daily activities affect not just the physical, but also the psychological, social, and spiritual aspects of patients’ lives, impacting their overall well-being (21). It is also common for HF patients to have chronic obstructive pulmonary disease due to shared risk factors, making them more fragile and limited than if they only had one condition (22). Encouraging these patients to join rehabilitation programs is crucial because physical training can boost their maximum oxygen uptake and exercise tolerance, and improve heart and cardiovascular function (23). A study by Reddy et al. found that poorer QoL was linked to reduced physical capacity and activity levels, rather than natriuretic peptide levels or resting heart function (24).

Poor QoL is also linked to a higher risk of adverse outcomes. According to a study by Ly et al., the risk of one-year adverse events, such as death, heart transplantation, re-hospitalization for acute HF, and mechanical circulatory support implantation, was significantly higher among adult congenital heart disease and HF patients with low physical functioning and poorer general health (25). Within one year, a significant portion of patients (14%) reached the combined endpoint, primarily due to re-hospitalizations for acute HF. In addition to poor QoL, patient comorbidities remained the most significant risk factors for severe cardiac events in these patients (25). Similarly, Zamora et al. reported a significant association between poor QoL and outcomes in HF with improved ejection fraction (HFimpEF). Patients with HFimpEF experienced greater improvements in QoL compared to those without HFimpEF (8). In a prospective cohort study by Wohlfahrt et al., normalization of heart function was linked to significant improvements in HF-specific QoL, better physical function, satisfaction with social roles, and reduced fatigue. Furthermore, a 10% increase in left ventricular ejection fraction was associated with a mean 4.8-point increase in the KCCQ-12 overall summary score among patients with HFrEF (26). A study by Pokharel et al. reported that regular monitoring with KCCQ can provide updated prognostic information for all HF patients, which can guide treatment decisions (27).

Several nonpharmacological, surgical, and medical interventions have been proposed that can help sustain or improve QoL in HF patients. Beyond cardiac rehabilitation and exercise training (28), other nonpharmacological interventions, such as treating depression and self-care interventions, can significantly improve QoL in HF patients. Among patients with HF, depression has been reported to be associated with an increased risk of adverse outcomes, including death and re-hospitalization (29). These patients are more likely to experience anxiety, mood disorders, and depression (30). Screening for depression through standardized screening pathways in HF patients could allow for early diagnosis and optimal treatment of depression, thereby improving QoL and outcomes (25). Furthermore, international guidelines, including the 2012 European Guidelines on cardiovascular disease prevention, recognize cardiac rehabilitation programs (CRPs) as a cost-effective, class I, level B intervention that improves prognosis, reduces hospitalizations and healthcare costs, and enhances QoL following acute coronary events (31). Although our study did not assess the uptake or impact of CRPs, these programs should be emphasized as an essential component of comprehensive heart failure management. Integrating CRPs more broadly in low- to middle-income settings like ours may offer a practical pathway to improving QoL among patients with heart failure.

It is important to recognize a few limitations of this study. First, it was conducted at a single center and was a descriptive cross-sectional study, meaning it only captured a snapshot of patients’ QoL at one point in time. Because of this, we cannot make broad generalizations or observe changes over time. Additionally, the study’s sample size was relatively small, which may limit the applicability of the findings to all heart failure patients, especially those in different regions or healthcare settings.

Moreover, since the study was cross-sectional, we could not assess the long-term effects of QoL on outcomes like mortality, hospitalizations, or disease progression. To truly understand how QoL impacts these long-term outcomes, more extensive and diverse studies are needed. Longitudinal studies, which follow patients over time, would provide a clearer picture of how improving QoL can contribute to better long-term health and well-being for heart failure patients.

## Conclusion

We observed good QoL in only one-fourth of the patients living with HF. Further, we observed that older age (>70 years), presence of diabetes, and non-adherence to medication were significantly associated with poorer QoL. These findings highlight the importance of focusing on specific areas to help HF patients lead better lives. While our findings highlight an association between medication adherence and better QoL, we caution against interpreting this as a causal relationship due to the study’s cross-sectional design. Nonetheless, these findings suggest that improving medication adherence could be an important focus for future research and interventions aimed at enhancing QoL in HF patients. Additionally, providing special support for older adults with heart failure is essential to lessen the negative impact on their daily lives. Overall, this study shows that treating heart failure should involve more than just addressing the medical aspects; it is about improving the overall well-being of patients and helping them enjoy a better QoL.

## Data Accessibility Statement

The datasets generated and/or analyzed during the current study are not publicly available due to institutional database privacy restrictions, but are available from the corresponding author on reasonable request.
